# Evaluation of Distolingual Canal/Roots in Mandibular Molars and Mesiobuccal Canals in Maxillary Molars by CBCT

**DOI:** 10.2174/1874210601711010360

**Published:** 2017-06-30

**Authors:** Filiz Namdar Pekiner, M. Oğuz Borahan, Asım Dumlu

**Affiliations:** Department of Oral Diagnosis and Radiology, Faculty of Dentistry, Marmara University, İstanbul, Turkey

**Keywords:** CBCT, Endodontic treatment, Root canal, Molar, Mesiobuccal Canal

## Abstract

**Background/Purpose:**

The objectives of this study were to identify the bilateral distolingual (DL) canals / roots of the mandibular first molars and second mesiobuccal (MB2) canals of the maxillary first molars in the same Turkish individuals using cone-beam computed tomography (CBCT).

**Materials and Methods:**

A total of 150 CBCT images including all mandibular and maxillary first molars were retrospectively investigated in a Turkish subpopulation. The patient age, sex and presence of roots and root canals were assessed. The frequency, of bilateral DL canals, DL roots, and MB2 canals were reviewed. Data were analyzed using Fisher’sexact test and *Chi*-square test.

**Results:**

The prevalences of right DL canals, DL roots and MB2 canals were 31.3, 14.0 and 34.7%, respectively. The prevalences of left DL canals, DL roots and MB2 canals were 31.3, 4 and 27.3%, respectively. There was no statistically difference in the frequency of right and left DL canals, DL roots of mandibular first molars and MB2 canals of maxillary first molars according to gender.

**Conclusion:**

CBCT is a competent tool for the detection of additional distolingual canals/roots and second mesio buccal canals, and it is a valuable aid for dentists providing root canal treatment.

## 1 INTRODUCTION

Success of endodontic management depends on the accurate debridement of the root canal space followed by complete obturation for obtaining the three-dimensional seal [1, 2]. Especially in multirooted teeth, root and root canal morphology variations are a constant challenge for management [3]. Therefore, a comprehensive knowledge of the root canal anatomy and its morphological variations is essential for successful treatment [2].

The mandibular first molars is known to display the most complex root and root canal morphology of the mandibular dentition [4]. Therefore many studies have reviewed the anatomy of the root canals of the mandibular first molars, and noted both complex anatomic variations and abnormalities [5, 6]. In these studies, the presence of a distolingual (DL) root of the mandibular first molars was reported to be 21.09-33.33% in various populations [7, 8]. However, it is reported that the presence of second mesiobuccal (MB2) canals of maxillary first molars is noticeably rare [6].

CBCT provides three-dimensional information of maxillofacial region and is increasingly being used in many of the dental specialties [8]. In terms of three-dimensional evaluation, two-dimensional imaging like periapical radiography and panoramic radiography loses the battle for the lack of perception [3,9]. Anatomical variations in the anatomy of the distal root/canal of mandibular molars and mesiobuccal (MB) canal of maxillary first molars may be determined through careful evaluation with the aid of multiple angled pretreatment radiographs. A two-dimensional image is obtained by conventional radiographs and they do not always reveal the actual number of roots and canals present in cases [10]. Recently, CBCT has been used in endodontics for the evaluation of the root canal morphology. An advantage of the CBCT imaging over the conventional radiograph is that it is proved to be a valuable technique for the diagnosis and evaluation of root canal anatomy [9, 11, 12]. Neelakantan *et al*. Found that CBCT was as accurate in identifying root canal systems as the modified canal staining [13]. Likewise, Tu *et al*. Showed a higher prevalence of extra roots in the mandibular first molars assessed by CBCT when compared with conventional radiography [14].

The aims of this study were to identify the bilateral distolingual (DL) canals / roots of the mandibular first molars and second mesiobuccal (MB2) canals of the maxillary first molars in the same Turkish individuals using cone-beam computed tomography (CBCT).

## 2 MATERIALS AND METHODS

### 2.1 Patient Data

This retrospective study sample is consisted of 150 patients (77 female, 73 male; 21-55 years old, mean 27.66 ± 6.08 years) who visited the department of Oral Diagnosis and Radiology at Dentistry Faculty and had CBCT scans for different purposes. 150 cone beam CT examinations of these patients with large FOVs showing all the mandibular and maxillary first molars fully erupted, formed apices and lacked root canal fillings, posts, and crown restorations, were picked up from the PACS (Picture Archiving Communication Systems) between 2012-2013 and were included in this study. CBCT was performed on a Planmeca Promax 3D Mid (Planmeca Oy, Helsinki, Finland). The ProMax 3D Mid CBCT machine was operated at 84 kVp and 4 mA with an 16×16 cm FOV, with the voxel size was 0.2 mm. The CBCT assessment was performed directly on monitor screen (Monitor 23 inch acer 1920x1080 pixel HP Reconstruction PC, USA). All of the included mandibular and maxillary first molars had completely form root apices and lacked root canal fillings, post, and crown restorations.

 The exclusion criteria included patients with history of trauma and/or surgery involving the maxillofacial region, systemic diseases which affect growth and development, or clinical and/or radiographic evidence of developmental anomalies/pathologies affecting the maxillofacial region. All digital imaging were viewed in the axial plane by Romexis 2.92 software (Planmeca Oy, Helsinki, Finland).

The study was carried out according to the recommendations of the Helsinki declaration and the study protocol was approved by the Local Committee of Research and Ethics of Yeditepe University (Protocol No: 212).

### 2.2 Observer

**Fig. (1):**
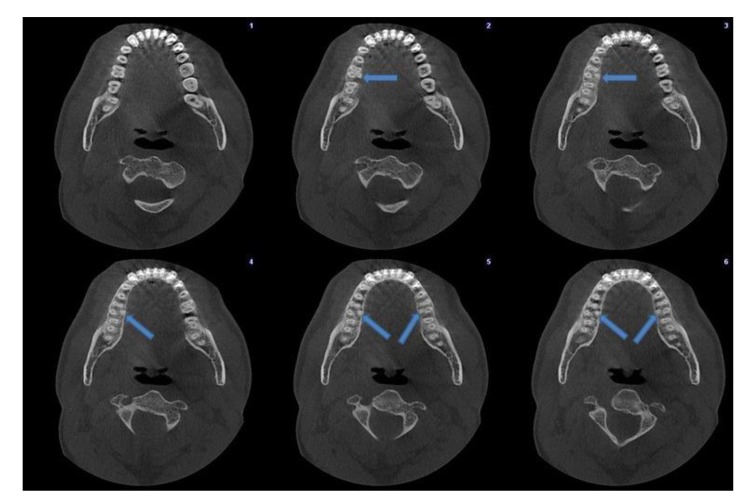
Axial sections showing bilateral mandibular first molars with DL roots.

**Fig. (2):**
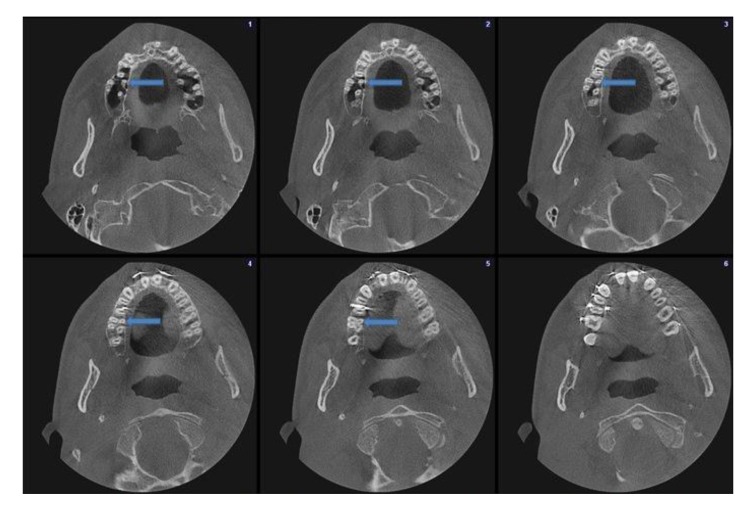
Axial sections showing right maxillary first molar with an MB2 canal and a left maxillary first molar without an MB2 canal.

An oral and maxillofacial radiologist (FNP) interpreted all images. Firstly, the assesment of patient age, gender and presence of roots and canals were performed. Secondly, the presence of DL roots and DL canals in the 150 right and 150 left mandibular first molars and their bilateral concurrence were recorded. If a DL root or DL canal was clearly separated from the distobuccal root or distobuccal canal, respectively, their presences were defined on the axial plane images (Fig. **1**). Lastly, the presence of an MB2 canal in the 150 right and 150 left maxillary first molars was recorded if it was clearly separated from main MB canal (Fig. **2**).

### 2.3 Statistical Analysis

The data were analysed with SPSS (Statistical Package for Social Sciences) for Windows 15.0. (SPSS Inc., Chicago. IL., USA). Descriptive statistical methods (mean, standard deviation, frequency) were used for the evaluation of the data. Chi-square test, Continuity (yates) Correction and Fisher’s exact test were used to evaluate the comparison of qualitative data. *P* values of less than 0.05 were interpreted as significant.

## 3 RESULTS

A total 300 mandibular first molars and 300 maxillary first molars were included in the study sample since every subject had mandibular and maxillary first molars on both sides. The prevalences of DL roots, DL canals of mandibular first molars, and MB2 canals of maxillary first molars were 11.3, 62.6, and point 6.0%, respectively.

The distributions of right DL canals, DL roots and MB2 canals were 31.3 14.0 and 34.7%, respectively. The distributions of left DL canals, DL roots and MB2 canals were 31, 3, 4.0 and 27.3%, respectively. There was no statistically difference in the frequency of right and left DL canals, DL roots of mandibular first molars and MB2 canals of maxillary first molars according to gender (Table **1**).

In Tables (**2** - **4**), the prevalences of the bilateral distolingual (DL) roots / canals of the mandibular first molars and second mesiobuccal (MB2) canals of the maxillary first molars are shown respectively. The rates of bilateral concurrence were significant for all variables *p* < 0.01.

## DISCUSSION

CBCT imaging technique was used in this study to evaluate the presence of mandibular first molars having distolingual roots/canals and second mesiobuccal canals of the maxillary first molars .

The prevalence of DL roots of mandibular first molars in this study was 11.3%, which was similar to those in previous reports [15, 16]. In addition, Scafer *et al*. Observed that the overall incidence of patients with three-rooted mandibular first molars was 1.35% and rare in a German population [17]. In contrast, the prevalence of DL roots of mandibular first molars was assesed by Kim *et al.* and it was reported that 24.7% had DL roots which was higher than our results [6]. Their result was similar to previous reports in Korean population with Mongolian traits [18, 19]. In other study, Tu *et al*. Determined the high prevalence of the DL root in mandibular first molars among the Taiwanese population [14]. In the present study, the prevalences of DL roots of mandibular first molars were lower in Turkish patients compared with Mongolian traits confirming previous findings that its incidence has been linked to specific ethnic groups [4, 6].

The percentage of DL roots of mandibular first molars was higher on the right side (14%) than on the left side (4%), and there was a siginifcant difference in this present study. In addition, the results of our study indicated a higher rate of unilateral prevalence of DL roots of mandibular first molars. Tu *et al.* and Song *et al.* also have observed right side predominance for DL roots of mandibular first molars [18, 20]. On the contrary, some studies showed that DL roots of mandibular first molars occur more frequently on left side [21, 22]. In other study, Miloğlu *et al.* [4] observed that the ratio of right/left side was quite similar, and the authors explained this incoordination could have resulted from variations in the populations, sample size, case selection and methods used.

Similarly, like in other studies, our study portrayed that there were no significant differences between the female and male subject for DL roots of mandibular first molars [23 - 25].

Although as many as five canals and as few as one and two canals occasionally occur in mandibular molars, the presence of four canals is relatively frequent [21, 26, 27]. In this study, the prevalence of DL canals of mandibular first molars was 31.3% on the left side and 31.3% on the right side, which was similar to the results of previous studies [6, 15]. In addition, similar with other studies there was not a significant difference in incidence of distolingual canals according to gender of patients.

There are many variations in canal number and configuration in maxillary first molar [28, 29]. Therefore, the authors stressed that failure to detect, debride, and fill a second mesiobuccal canal (MB2) of first permanent maxillary molars was one of the main causes of poor long-term prognosis after root canal treatment in these teeth [26]. According to the results of our study, there was not a significant difference in incidence of MB2 canals in terms of gender of patients and the prevalence of MB2 canals of maxillary molars falls within the range of these previous studies. The study protocols (*in vivo* or *in vitro*) and the techniques used to identify canal configuration may lead to differences observed between these studies.

CBCT scanning has been used in endodontics for the efficient evaluation of the root canal morphology although intraoral radiographs still remain the imaging technique of choice for the evaluation of endodontic patients. CBCT with relatively low patient doses and small voxel sizes should be preffered for diagnosis in patients who present with contradictory or non-specific clinical signs and symptoms associated with untreated or previously endodontically-treated teeth [30]. The position paper published jointly by the American Association of Endodontists (AAE) and the American Academy of Oral and Maxillofacial Radiology (AAOMR) does not support the routine use of CBCT for all cases except when complex root canal anatomy is suspected [31].

To determine variations in root canal morphology, various techniques have been suggested: conventional and modified tooth staining and clearing, conventional and digital radiography, contrast media radiography and computed tomography (CT). CBCT has a superiority over conventional periapical films due to its ability of to reduce or eliminate superimposition of surrounding structures [32]. Therefore, CBCT is the best imaging technique for the evaluation and identification of additional root/canals. For this reason, we evaluated these root/canal anatomic variations by using CBCT from axial sections.

## CONCLUSION

Dentists should be familiar with root canal morphology and should be aware of unexpected canal morphology when performing endodontic treatment. Also, clinicians should carefully observe CBCT scans of patients justified for other reasons to investigate the presence of extra root/canal. The present report portrayed the use of a cone-beam computed tomography examination as a tool for the diagnosis and negotiation of extra canals in the distal root/canals of mandibular first molars and second mesibuccal canals of maxillary first molars. Information gained through this type of studies will be used in the future for diagnosis and endodontic therapy which may contribute to aid clinicians in the prediction of additional canals.

## Figures and Tables

**Table 1:** Distribution of right and left DL canals, DL roots of mandibular first molars and MB2 canals of maxillary first molars according to gender.

	**Male (n=73)**	**Female (n=77)**	**Total (n=150)**	***p***
	**n (%)**	**n (%)**	**n (%)**	
**^+^36 DL Root**	2 (2.7%)	4 (5.2%)	6 (4.0%)	**0.682**
**^++^46 DL Root**	9 (12.3%)	12 (15.6%)	21 (14.0%)	**0.566**
**^++^36 DL Canal**	19 (26.0%)	28 (36.4%)	47 (31.3%)	**0.173**
**^++^46 DL Canal**	18 (24.7%)	29 (37.7%)	47 (31.3%)	**0.086**
**^++^16 MB2 Canal**	20 (27.4%)	21 (27.3%)	41 (27.3%)	**0.986**
**^++^26 MB2 Canal**	26 (35.6%)	26 (33.8%)	52 (34.7%)	**0.812**

^+^ Fisher’s Exact test ++Chi-square test

**Table 2:** Evaluation of the relationship between 36 DL root and 46 DL root.

**46 DL Root**	**36 DL Root**			***p***
	**Presence**	**Absence**	** Total **	
	**n (%)**	**n (%)**	** n (%) **	
**Presence**	4 (2.7%)	17 (11.3%)	21 (14.0%)	0.004**
**Absence**	2 (1.3%)	127 (84.7%)	129 (86.0%)	
**Total**	6 (4.0%)	144 (96.0%)	150 (100%)	

Fisher’s Exact test ** *p*<0.01

**Table 3:** Evaluation of the relationship between 36 DL canal and 46 DL canal.

**46 DL Canal**	**36 DL canal**			***p***
	**Presence**	**Absence**	** Total **	
	**n (%)**	**n (%)**	** n (%) **	
**Presence**	30 (20.0%)	17 (11.3%)	47 (31.3%)	0.001**
**Absence**	17 (11.3%)	86 (57.3%)	103 (68.7%)	
**Total**	47 (31.3%)	103 (68.7%)	150 (100%)	

Continuity (yates) Correction ** *p*<0.01

**Table 4:** Evaluation of the relationship between 16 MB2 canal and 26 MB2 canal.

**26 MB2 Canal**	**16 MB2 canal**			***p***
	**Presence**	**Absence**	** Total **	
	**n (%)**	**n (%)**	** n (%) **	
**Presence**	28 (18.7%)	24 (16.0%)	52 (34.7%)	0.001**
**Absence**	13 (8.7%)	85 (56.7%)	98 (65.3%)	
**Total**	41 (27.3%)	109 (72.7%)	150 (100%)	

Continuity (yates) Correction ** *p*<0.01
